# Next step trauma and orthopaedic surgery: integration of augmented reality for reduction and nail implantation of tibial fractures

**DOI:** 10.1007/s00264-022-05619-3

**Published:** 2022-11-15

**Authors:** Tim Klopfer, Thomas Notheisen, Heiko Baumgartner, Dorien Schneidmueller, Ryan Giordmaina, Tina Histing, Christoph Emanuel Gonser

**Affiliations:** 1 Orthopaedische Chirurgie Bayreuth, Bayreuth, Germany; 2 BG Trauma Center Tuebingen, Trauma and Reconstructive Surgery at the University of Tuebingen, Schnarrenbergstr. 95, 72076 Tuebingen, Germany; 3 BG Trauma Center Tuebingen, Trauma and Reconstructive Surgery, Murnau, Germany; 4 Department of Trauma and Orthopaedics, Mater Dei Hospital, University of Malta, Msida, Malta

**Keywords:** Intra-operative imaging, Intra-operative 3D imaging, Augmented reality in surgery, Navigation, Head-mounted display

## Abstract

**Introduction:**

There is a tremendous scope of hardware and software development going on in augmented reality (AR), also in trauma and orthopaedic surgery. However, there are only a few systems available for intra-operative 3D imaging and guidance, most of them rely on peri- and intra-operative X-ray imaging. Especially in complex situations such as pelvic surgery or multifragmentary multilevel fractures, intra-operative 3D imaging and implant tracking systems have proven to be of great advantage for the outcome of the surgery and can help reduce X-ray exposure, at least for the surgical team (Ochs et al. in Injury 41:1297 1305, 2010). Yet, the current systems do not provide the ability to have a dynamic live view from the perspective of the surgeon. Our study describes a prototype AR-based system for live tracking which does not rely on X-rays.

**Materials and methods:**

A protype live-view intra-operative guidance system using an AR head-mounted device (HMD) was developed and tested on the implantation of a medullary nail in a tibia fracture model. Software algorithms that allow live view and tracking of the implant, fracture fragments and soft tissue without the intra-operative use of X-rays were derived.

**Results:**

The implantation of a medullar tibia nail is possible while only relying on AR-guidance and live view without the intra-operative use of X-rays.

**Conclusions:**

The current paper describes a feasibility study with a prototype of an intra-operative dynamic live tracking and imaging system that does not require intra-operative use of X-rays and dynamically adjust to the perspective of the surgeons due to an AR HMD. To our knowledge, the current literature does not describe any similar systems. This could be the next step in surgical imaging and education and a promising way to improve patient care.

## Introduction

There is a tremendous scope of hardware and software development going on in augmented reality (AR). This development also spreads into medicine; however, there are only a few established applications in use so far, especially as for trauma surgery. This also applies to intra-operative imaging and guidance. There are some systems available for intra-operative 3D imaging and guidance, most of them rely on peri- or intra-operative imaging deploying X-rays. Trauma surgeons are often faced with complex situations which arise intra-operatively, might it be in emergency procedures or complex elective cases. Especially in these complex situations such as pelvic surgery or multifragmentary multilevel fractures, intra-operative 3D imaging and implant tracking systems have proven to be of great advantage for the outcome of the surgery and can help reduce X-ray exposure, at least for the surgical team [1]. Moreover, intra-operative dynamic life tracking and imaging can help improve and facilitate surgical education. However, there is still space for improvements and further development. Although currently available imaging devices deliver excellent static 3D images. Yet, these methods do not provide the ability to have a dynamic live view from the perspective of the surgeon. In complex situations such as multifragmentary fractures the fit of the implant and the fragments must be determined intra-operatively; this is mostly done by X-ray imaging, regardless of 2D or 3D. Moreover, these methods do not show the perspective as the surgeon sees the fracture site.

To our mind, intra-operative AR-enhanced imaging using overlays of pre-operatively gathered CT or MRI scans on to the site greatly improves the knowledge of the orientation of the different fragments and the implant. This can lead to reduced X-ray exposure and faster procedures with better results.

The aim of this study was to develop a system that allows an intra-operative overlay of different layers of the site starting with the skin up to the bone using a head mounted display at the example of closed reduction and nailing of a tibial fracture. Moreover, we examined if AR technologies can help to make surgical procedures safer and deepen the understanding of different intra-operative situations. We wanted to investigate if an AR device can be used under real-world conditions in the OR and be controlled with a virtual user interface while wearing sterile surgical gloves. Moreover, we wanted to examine if closed reduction and implant placement is possible without the use of X-rays just relying on the AR guidance und real-world conditions in the OR using a realistic sawbone model.

## Materials and methods

For the prototype, we used the latest generation AR-HMD of Microsoft’s HoloLens (HoloLens 2, Microsoft Cooperation, Redmond, WA, USA). This device fulfills all the stated prerequisites. The conceptual idea and as well as the model were developed by the authors, additional coding support was provided by AmbiGate Motion Sensing GmbH, Tuebingen, Germany. First of all, a model consisting of an artificial tibia bone with a multifragmentary fracture and artificial soft tissue (Sawbones, A Pacific Research Company, Vashon Island, Washington, USA) forming a model of a lower leg was fitted with an external fixator (DepuySynthes, Umkirch, Germany).

The tibia fracture was chosen for the following reasons:

- Tibia fractures are rather common and closed reduction and nail osteosyntheses is an established treatment [2].
- The successful treatment depends on the exact entry point and correct reposition as well as axis reconstruction [3].
- Closed reduction and insertion of the nail into the core canal of the distal fragment can be quite demanding and require several tries and multiple X-ray images resulting in a high X-ray exposure for the surgical team and patient [4].

In this situation, the AR augmented live tracking can provide significant advantages. To be able to use this technique intra-operatively, we defined the following prerequisites:

1. The AR glasses must not disturb or restrict the surgeon.
2. Sterility must absolutely be guaranteed.
3. The system must be easy to use with a clean user interface (UI).
4. Direct visualization of the positional relation of the different fragments with high precision and low latency of the overlay CT/MRI data.
5. The system must allow customizable 3D visualization with adjustable views as for zoom, rotation, position, and transparency.
6. The surgeon should be able to select different layers of the segmented CT/MRI.
7. Simultaneous view of the AR Phantom with bone and implant overlayed on the site.
8. Live view of the reduction of the bone fragments.
9. Implementation of recording and casting functions (audio, video).

**Fig. 1:**
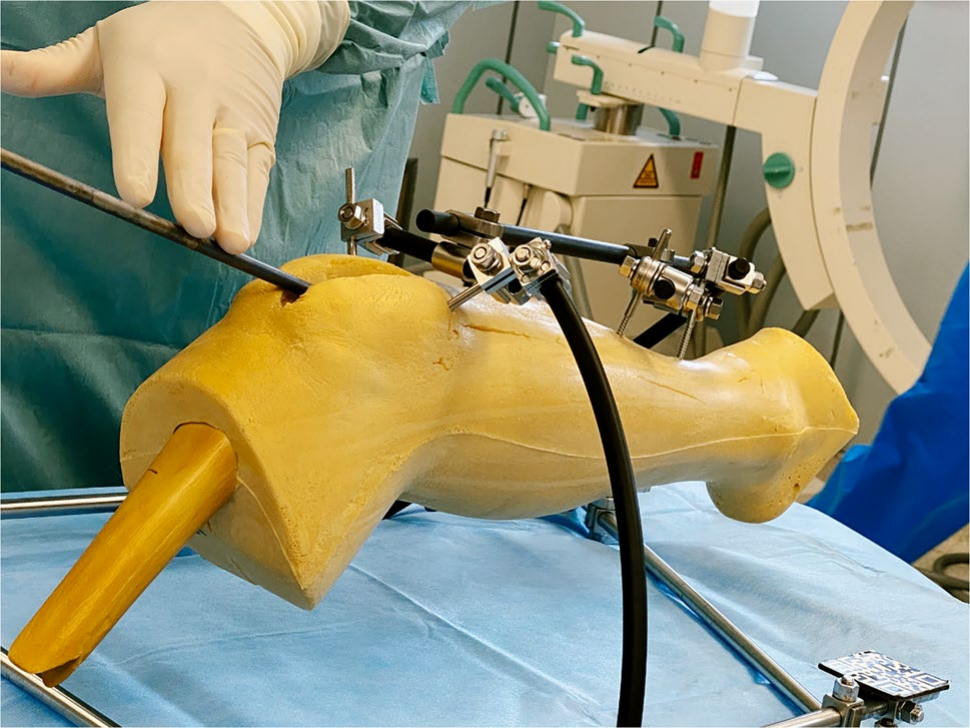
The model consisting of a sawbone lower leg with soft tissue and an external fixator with attached QR codes

This model (see Fig. 1) was then scanned with a 64-line CT scanner (Siemens Health Care, Erlangen, Germany) using a protocol with 120 kV, 90 mAs, 0.75 mm slice thickness. In a second step, a regular tibia nail including the aiming arm (DepuySynthes, Umkirch, Germany) which is also used in clinical routine, was scanned with the same protocol.

These data were than segmented using Mimics Innovation suite (Materialise GmbH, Munich, Germany). This provided separate high-quality three-dimensional image of all relevant structures:

1. Tibia nail
2. Aiming arm
3. External fixator
4. Soft tissues and skin
5. Bone

**Fig. 2:**
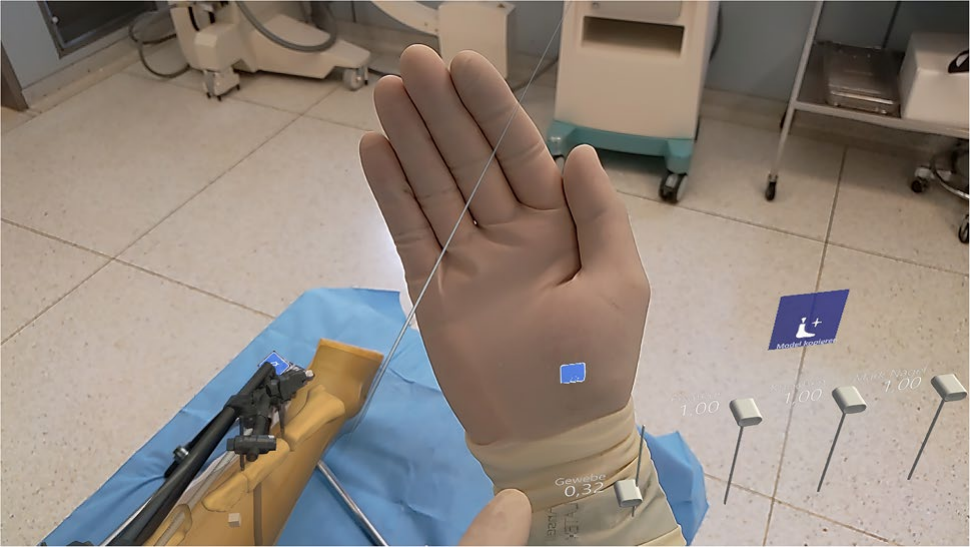
The virtual user interface with sliders to adjust the opacity of each layer seen in the lower right corner

**Fig. 3:**
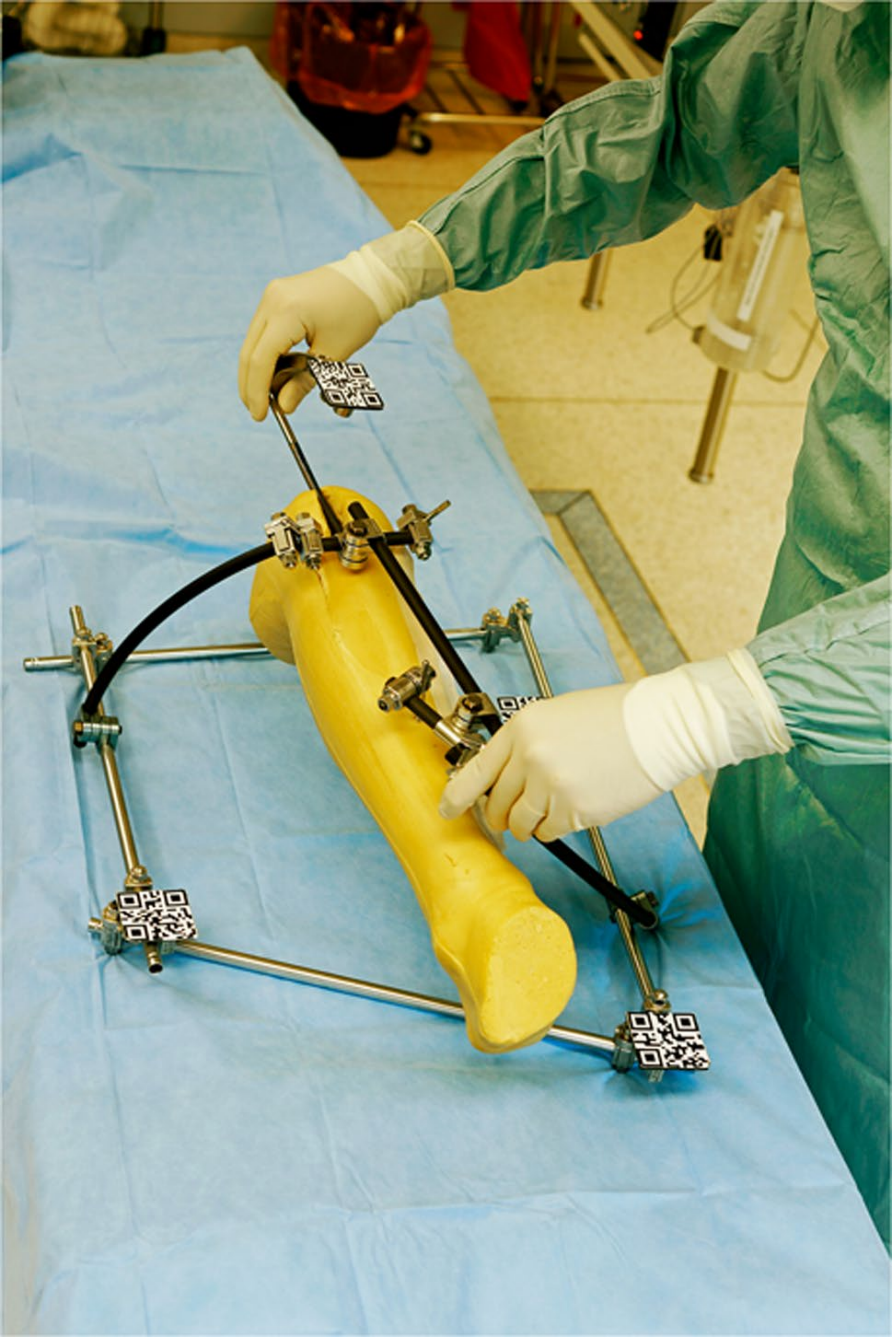
QR codes used to identify the different parts of the model

In order to project the data on the site, a user interface (UI) was developed which allow to live-visualize the implantation of the tibia nail as a hologram of the segmented data in all planes in space. Figure 2 shows part of the virtual user interface. We used QR codes which were applied to the external fixator and the implant (Fig. 3) to track these parts. We developed an algorithm that allowed the HoloLens2 AR-HMD to precisely locate the marked elements and visualize them in physical space and project the overlay to the site without the use of additional X-ray imaging. The algorithm was refined to optimized precision and latency and to offer additional virtualization options:

With the prototype finished to the abovementioned specifications, the following scenarios were tested in the OR using the phantom:

- Use of the system under real life conditions in the operating room: The phantom was placed on an operating table, sterile draping was applied after disinfection, sterile surgical gowns were used to assess the impact of the AR-HMD on the surgeon and to test operationality of the system and the UI.
- The quality of the reduction and the implant position were visually assessed using a medial incision on the model (Fig. 6).
- Closed reduction and nail insertion relying only on the AR-HMD were tested.
- The accuracy of the overlay was evaluated.
- The usability and operationality of the system regarding OR lighting conditions.

**Fig. 4:**
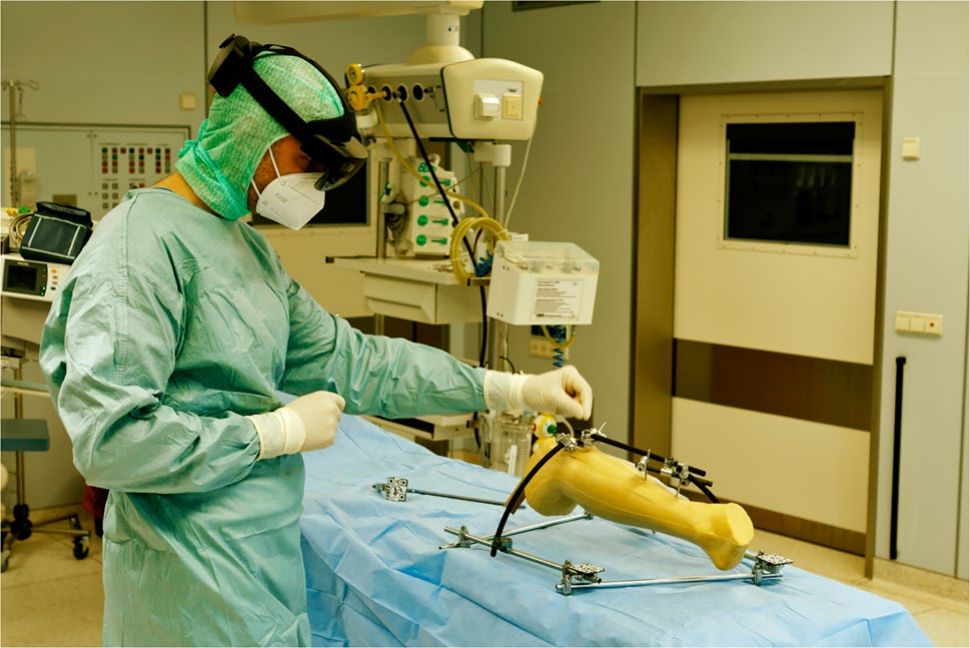
Intra-operative use of the system with the surgeon performing different gestures to zoom/rotate the virtual model in the physical space

**Fig. 5:**
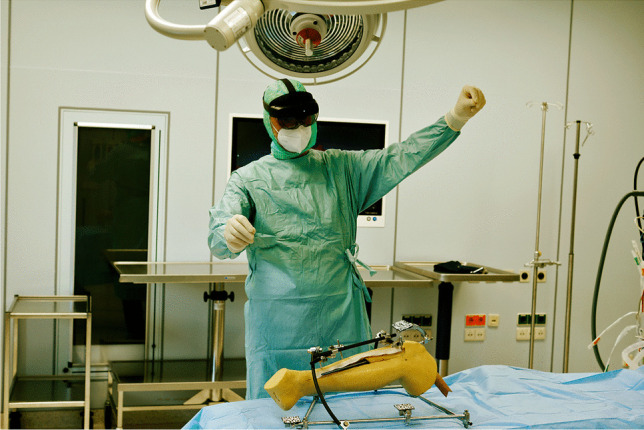
Intra-operative use of the system with the surgeon performing different gestures to zoom/rotate the virtual model in the physical space

The tests were performed by four surgeons in a set of 5 runs a day using a rotation system so that each surgeon had a break between the individual implantations to reduce a habituation effect. Altogether, 40 runs were performed. Figures 4 and 5 show the intra-operative use of the system.

## Results

While using the prototype under OR conditions, the user interface (UI) was easy and comfortable to use with an intuitive control set.

**Fig. 6:**
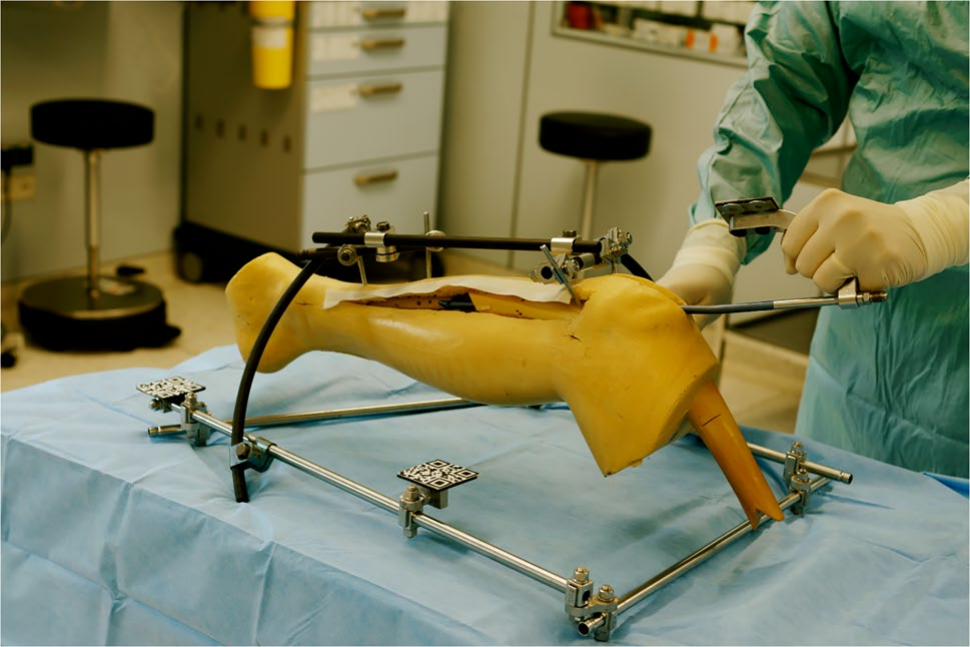
Medial incision to manually check the reduction and implant placement

The correct insertion of the nail into the tibia using only AR guidance was achieved reproducibly. In all 40 test implantations, a correct implant position with an anatomical reduction was achieved relying only on the AR navigation (Fig. 6).

The system showed some deviation of the overlay of the phantom to the site under severe head movements of the surgeons, these could be corrected by recalibrating the system intra-operatively by scanning the QR codes.

**Fig. 7:**
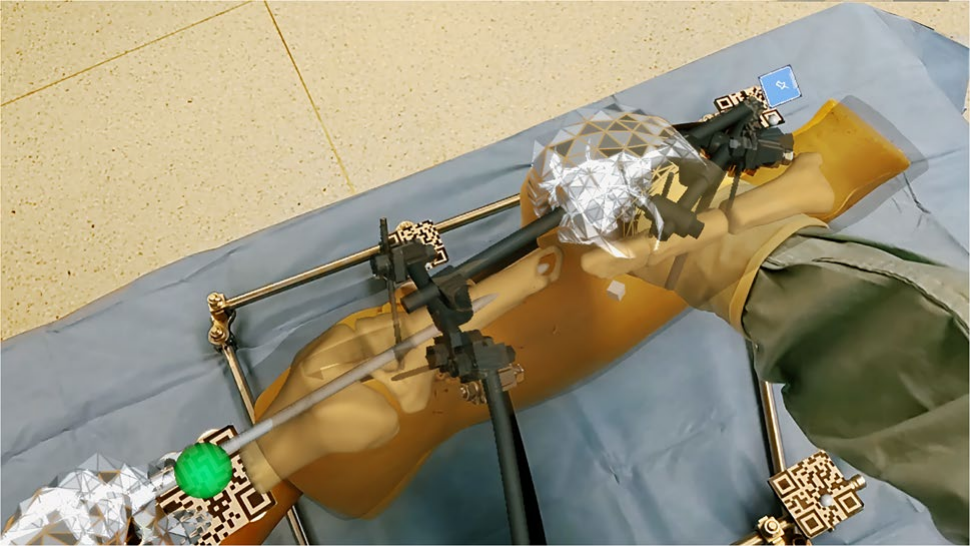
Image of the overlay of the implant and bones of the virtual model on the real site

The three-dimensional visualization and overlays were deemed excellent by all four users. All four users stated that the AR-augmented visualization delivered valuable information and facilitated the process. Figure 7 shows a sample image of an overlay on the site with the user interface of the AR-HMD.

None of the test users found the system to negatively affect the surgeon’s comfort, sterility, or liberty of movement.

The visualization options were assessed excellent by the test users. However, during test implantation, several cases of loss of the tracking of the nail occurred. This happened when the QR codes of the aiming arm disappeared from the field of view and is attributed to the movement of the head of the surgeon.

The changing lighting conditions in the OR with high contrast scenarios did not cause any problems; however, two cases of reduced picture quality of the HMD were reported when the HMD was in the direct ray of light of a surgical light.

Last, the added functionality of showing the virtual OP Site of the phantom in any random position in space with any zoom level as a hologram was tested. This functionality was assessed as very useful by three surgeons, one surgeon assessed it as useful.

Due to the wireless construction of the HMD, sterility was not compromised in the usage scenario. The tracking of the surgeon’s hand and the operation of the virtual user interface were not negatively affected using sterile surgical gloves.

To check how easy and intuitively the system is operable, we defined the core prerequisites:

1. The AR glasses must not disturb or restrict the surgeon.
2. Sterility must absolutely be guaranteed.
3. The system must be easy to use with a clean user interface (UI).
4. Direct visualization of the positional relation of the different fragments with high precision and low latency of the overlay CT/MRI data.
5. The system must allow customizable 3D visualization with adjustable views as for zoom, rotation, position, and transparency.
6. The surgeon should be able to select different layers of the segmented CT/MRI.
7. Simultaneous view of the AR Phantom with bone and implant overlayed on the site.
8. Live view of the reduction of the bone fragments.
9. Implementation of recording and casting functions (audio, video).

All of the abovementioned prerequisites were met with the exemption of prerequisite 4: several loss of tracking incidences occurred under severe head movements of the surgeon and precision of the overlay was reduced during very fast head movements.

## Discussion

Up to date, to our knowledge, no other comparable feasibility study for the AR guided implantation of a tibia nail in a real-world scenario [5, 6, 7] although fractures of the lower leg are commonly found in all age clusters [2]. AR integration in surgical procedures still seems to be rare, although we think there is a tremendous potential to be unleashed. Some workgroups investigated the implantation of K-wires into the pelvis or pedicles of the vertebra. In a cadaver model, Wang et al. could show that placing IS-Joint screws with the help of AR is possible with minimal deviation and without lesions to vessels or nerval structures [8]. Ochs and Gonser demonstrated that with the help of three-dimensional navigation, radiation exposure could be reduced [9]. A reduction in radiation dose is very important, since especially in these procedures, radiation doses experienced by the surgical team are high [10]. Studies combining AR and a conventional C-arm X-ray device showed promising results as for the reduction X-ray exposure and procedure duration [11, 12]. This was also found by Fischer when placing K-wires into different models [13]. Gibby et al. used the HoloLens1 for pedicle screw placement and found only minimal misalignment compared to the placement with X-ray support. Liu et al. found only minimal deviation of an AR-guided placement of a K-wire for hip-resurfacing [14].

Hybrid models such as the integration of C-Arm images and sonographically acquired images have been tried as well: Hajek et al. have used a HoloLens and a C-Arm for percutaneous interventions [15]. Heide et al. could show that X-ray exposure could be reduced by 46% by using such a hybrid method [16].

All these findings concur with our findings showing that a high level of precision can be reached with AR-guided implant placement, even without the use of X-rays.

So far, we can conclude that there are several approaches to the use of AR in surgery. These approaches are on different evolutional levels. The described prototype opens a new perspective by using AR to guide the implantation of a tibia nail without the intra-operative use of X-ray.

To our opinion, a key factor for clinical use of AR technology in the OR is the ease of use. The described prototype was easy to use for all testers. One future development should be the implementation of smaller QR markers or even a markerless system—further development is necessary. Further use-scenarios can be found in endoprosthetics, e.g., for planning and live-tracking of component placement. Reliable results for placement of the acetabular component have already been found by Ogawa [17]. Further enhancements in precision are to be expected with the evolution of technology and corrections algorithms. Oliveria et al. could already show that reliability and precision were enhanced with added algorithms to tackle latency and orientation in physical space [18].

The benefit of being able to intra-operatively adjust the views as for angle and zoom as well as other parameters was already shown by Gregory et al. when overlaying a CT scan and a three-dimensional planning sketch during implantation of an inverse shoulder prothesis [19]. Ochs and Gonser could show that visualization of different levels and reference points facilitates complex procedures [20]. We could show that the developed user interface was easy to use in the OR und real-life conditions without compromising on sterility or patient safety. We could show that a safe integration into the surgical workflow is possible. The UI was found easy to use and all the testers confirmed that the ability to intra-operatively navigate through the projected hologram of the fracture freely in physical space helped to gain better insight. Some reviews show an increasing interest in the use of AR-technology in surgery, however there are still problems to be solved, e.g., motion sickness or precision of the overlay. But apart from all these problems, even the currently available systems were found to be able to reduce the degree of difficulty and the X-ray exposure [6, 7]. A further advantage can be found in the ability to actively interact with other experts on the field which are not physically present through teleconsultation with the possibility to interact [21]. This is also applied to neurosurgery: Incekara et al. showed that focusing on a tumor areal was eased with the help of AR with high precision overlays [1]. For such highly complex procedures, we think, at least as phase of transition, the combination of several imaging sources and intra-operative imaging would help to enhance safety. Vessel and important neural structure could precisely be shown in the overlay and through the tracking of the instruments, a feedback would be possible if the surgeons approaches too closely to one of these structures. Solely AR-guided complex surgery is a future perspective.

We found a high level of precision of the AR device which enabled us to precisely implant a tibia nail. However, there were some limitations: There were some cases of loss of tracking. We attribute this to a to some extent limited angle of view of the built-in cameras of the HMD. The limited field of view of the built in-cameras also limited the ability of the system to recognize hand gestures. Future technological development will probably also address this. Another limitation of our system is the need to mark all the relevant surfaces with QR markers. So far, all other systems described used some way of markers as well, another way is using a pattern of reflective cue points. Our focus right now lies on the development of a markerless system with enhanced precision. For clinical use in humans, the use of a backup X-ray system to verify implant positions seems inevitable at the current point. The future aim is a system that can be used as standalone device without intra-operative X-ray; this would not only reduce X-ray exposure for the patient and the surgical team but also the number of devices required in the OR.

A markerless system would also allow to overcome another limitation of the present system: not all boney fragments were marked and thus not live tracked during surgery. In our model, all major fragments were tracked with the attached external fixator. A system which will be able to track at least all fragments bigger than 1 cm^2^ is in development.

In our opinion, the presented system provides possibilities of intra-operative planning and step by step verification that is unpreceded; also, there are some limitations which have to be tackled in future development.

## Conclusions

The current paper describes a feasibility study with a prototype of an intra-operative dynamic live tracking and imaging system that does not require intra-operative use of X-rays and dynamically adjust to the perspective of the surgeons due to an AR head-mounted display (HMD). To our knowledge, this is the first description of such a system. Our study showed that that the implantation of a tibia nail in a real-life model is possible relying only on AR guidance. Excellent visualization of the pre-operatively gained image data was shown, the ability to free rotate the hologram in physical space and to zoom as well as the precise overlay onto the surgical site were highly rated.

We could prove that AR guidance works under real-life conditions in the OR without compromising sterility and that the system was easy to use even when sterilely dressed. Further development should improve precision and the ability to track small boney fragments. The development and use of hybrid models should be examined in further studies.

## Data Availability

All data is available.
